# Synaptonemal complex analysis of interracial hybrids between the Moscow and Neroosa chromosomal races of the common shrew *Sorex araneus* showing regular formation of a complex meiotic configuration (ring-of-four)

**DOI:** 10.3897/CompCytogen.v6i3.3701

**Published:** 2012-09-14

**Authors:** Sergey N. Matveevsky, Svetlana V. Pavlova, Maret M. Acaeva, Oxana L. Kolomiets

**Affiliations:** 1 N.I. Vavilov Institute of General Genetics, Russian Academy of Sciences, Gubkin str. 3, Moscow 119991, Russia; 2 A.N. Severtsov Institute of Ecology and Evolution, Russian Academy of Sciences, Leninskiy pr. 33, Moscow 119071, Russia; 3 Chehen State University, A. Sheripov str. 32, Grozny 364051, Chechen Republic, Russia

**Keywords:** Synaptonemal complex, MSCI, γH2AX, centromeres, *Sorex araneus*

## Abstract

Immunocytochemical and electron microscopic analysis of synaptonemal complexes (SCs) was carried out for the first time in homozygotes and complex Robertsonian heterozygotes (hybrids) of the common shrew, *Sorex araneus* Linnaeus, 1758, from a newly discovered hybrid zone between the Moscow and the Neroosa chromosomal races. These races differ in four monobrachial homologous metacentrics, and closed SC tetravalent is expected to be formed in meiosis of a hybrid. Indeed, such a multivalent was found at meiotic prophase I in hybrids. Interactions between multivalent and both autosomes and/or the sex chromosomes were observed. For the first time we have used immunocytochemical techniques to analyse asynapsis in *Sorex araneus* and show that the multivalent pairs in an orderly fashion with complete synapsis. Despite some signs of spermatocytes arrested in the meiotic prophase I, hybrids had large number of active sperm. Thus, Moscow – Neroosa hybrid males that form a ring-of-four meiotic configuration are most likely not sterile. Our results support previous demonstrations that monobrachial homology of metacentrics of the common shrew does not lead to complete reproductive isolation between parapatric chromosomal races of the species.

## Introduction

The concept of chromosomal speciation implies occurrence of reproductive isolation as a result of chromosomal rearrangements (Vorontsov 1960, White 1978, 1982, King 1993). The most common type of chromosome rearrangements in mammalian evolution is represented by the Robertsonian translocations – fusion of two acrocentric chromosomes into a single submeta – or metacentric chromosome. It was first described in orthopterous insects (Robertson 1916). Species with a so-called “Robertsonian fan” represent unique models for studying chromosomal speciation. The term was introduced by R. Matthey for description of a wide-range chromosomal variation caused by multiple Robertsonian translocations (Matthey 1970). Among mammals, there are several species that demonstrate the Robertsonian fan: the Subsaharan pygmy mouse, *Mus (Nannomys) musculoides* Temminck, 1853 (Matthey 1970, Jotterand 1972), the house mouse, *Mus musculus domesticus* Schwarz et Schwarz, 1943 (Gropp et al. 1972, Gropp and Winking 1981), the Eastern mole vole, *Ellobius tancrei* Blasius, 1884 (Lyapunova et al. 1980), the Nigerian gerbil, *Gerbillus nigeriae* Thomas et Hinton, 1920 (Volobouev et al. 1988), the Daghestan pine vole, *Pitymys daghestanicus* Shidlovsky, 1919(Tembotov et al. 1976), and the common shrew, *Sorex araneus* Linnaeus, 1758 (Searle and Wójcik 1998).

Due to its high level of karyotype variability, the common shrew *Sorex araneus* is subdivided into at least 72 parapatric chromosomal races, each characterised by a unique set of metacentric chromosomes formed by Robertsonian fusions and/or whole-arm reciprocal translocations (WARTs) (Hausser et al. 1994, White et al. 2010). Three metacentrics (*af*, *bc*, *tu*) and sex chromosomes (XX in females and XY_1_Y_2_ trivalent in males) are invariant in all chromosomal races, while another ten autosomal arms (*g-r*) may occur as acrocentrics and/or combined together as metacentrics (Searle et al. 1991). XY_1_Y_2_ sex trivalent formed by the X-autosome translocation (Sharman 1956) is specific for the species of the ‘*Sorex araneus*’ group (Zima et al. 1998). The sex trivalent has original parts (*e* “true” arm of X chromosome and the Y_1_) and autosomal parts (*d* translocated arm of X chromosome homologous to the Y_2_) (Fredga 1970, Pack et al. 1993).

Hybrids between parapatric chromosomal races of the common shrew are often expected to be complex Robertsonian heterozygotes with monobrachial homology, which form chain (C) or ring (R) configurations of three or more elements at prophase I of meiosis. Such complex meiotic configurationsare considered to be more susceptible to irregularity. As a consequence, complex heterozygotes are expected to be less fertile than homozygotes of pure chromosomal races. At present, interracial hybrids with different types of meiotic configurations from CIII and RIV up to CXI and RVI have been revealed from seventeen well-studied hybrid zones (Searle and Wójcik 1998, Bulatova et al. 2011, Polyakov et al. 2011, Orlov et al. 2012). Studies so far have shown that hybrids with long chain or ring configurations have more abnormalities during meiosis than hybrids with shorter configurations; however even in these cases the complex meiotic configurations do not appear to be associated with complete sterility (Mercer et al. 1992, Narain and Fredga 1997, Jadwiszczak and Banaszek 2006, Pavlova et al. 2008). There is a need to document more fully the match between complexity of karyotype and degree of regularity of the meiotic configurations expected.

A new chromosomal hybrid zone between the Moscow race (***gm***, *hi*, *jl*, *kr*, ***no***, *pq, 2n*a=18) and the Neroosa race (***go***, *hi*, *jl*, *kr*, ***mn***, *pq, 2n*a*=*18) has been found recently in the centre of European Russia (Pavlova et al. 2012, *in press*). Karyotypes of the races differ in four metacentrics with monobrachial homology so that the complex heterozygotes should form a ring-of-four (RIV) configuration at meiosis I. On the basis of karyotype differences of the races one can suggest that fixation of just one WART, between metacentrics *gm* and *no* or between *go* and *mn*, could have separated these races in the past.

This paper presents a comparative synaptonemal complex (SC)analysis of prophase I of meiosis using electron microscopy and immunofluorescence in homozygotes and complex Robertsonian heterozygotes from this hybrid zone. A combination of both methods together for SC analysis is used for the first time in *Sorex araneus*.

## Material and methods

***Animals and karyotypes*.** A total of eight adult male common shrews were collected from the Moscow-Neroosa hybrid zone, located in the south-eastern part of the Moscow Region near Ozyory town (the left bank of the River Oka), in April 2012, at the beginning of the breeding season. Each specimen was processed according to the field procedure described in Bulatova et al. (2009). Mitotic chromosomes were prepared from bone marrow and spleen following Ford and Hamerton (1956) with modifications. A trypsin - Giemsa staining technique of Kràl & Radjabli (1974) was used for identification of chromosome arms according to the standard nomenclature for the *Sorex araneus* karyotype (Searle et al. 1991). Only three of eight karyotyped males were used for the meiotic analysis.

***Synaptonemal Complex Analysis***. Synaptonemal complex (SC) preparations were prepared and ﬁxed using the technique of Navarro et al. (1981) with modiﬁcations (Kolomiets et al. 2010). Measurements of autosomal bivalents and their ranking in each cell were made in order to determining relative lengths (MicroMeasure 3.3, Colorado, USA).

***Electron microscopy*.** Slides were stained with a 50% AgNO_3_ solution in a humid chamber for 3 h at 56°C, washed 4 times in distilled water and air dried. Stained slides were observed under a light microscope to select suitably spread cells. Once selected, plastic (Falcon film) circles were cut out with a diamond tap and transferred onto grids and examined in a JEM 100B electron microscope.

***Immunofluorescence*.** Poly-L-lysine-coated glass was used for immunostaining. The slides were placed in phosphate-buffered saline (PBS) and incubated overnight at 4°C with the following primary antibodies diluted in antibody dilution buffer (ADB: 3% bovine serum albumin - BSA, 0.05% Triton X-100 in PBS): rabbit anti-SCP3 1:200 (Abcam,Ab15093), human anti-centromere antibodies, ACA 1:200 (Antibody Incorporated, 15-235) and mouse anti-phospho-histone γH2AX 1:500 (Abcam,Ab26350). After rinsing in PBS (3 times for 10 min), the slides were incubated with appropriate secondary antibodies diluted 1:800 in PBS: goat anti-rabbit Alexa Fluore 488 conjugated antibodies, goat anti-human Alexa Fluore 546 conjugated antibodies and FITC-conjugated horse anti-mouse IgG (all Abcam) at 37°C for 90 min. After a final rinse in PBS, the slides were mounted in Vectashield with DAPI (Vector Laboratories). Slides were analyzed in an Axioimager D1 microscope CHROMA filter sets (Carl Zeiss, Jena, Germany) equipped with an Axiocam HRm CCD camera (Carl Zeiss), and image-processing AxioVision Release 4.6.3. software (Carl Zeiss, Germany). Images were processed using Adobe Photoshop CS3 Extended.

## Results

**Karyotypes.** Three of the eight karyotyped shrews were complex heterozygotes, i.e. F1 hybrids. They showed the expected arm combinations of Rb metacentrics - *go/gm/mn/no, hi, jl, kr, pq*. Five other shrews were homozygotes with Moscow race karyotype (*gm, hi, jl, kr, no, pq*). Hybrid individuals and homozygotes of the pure race had 2n=21, NF=40, XY_1_Y_2_. Only two hybrids and one homozygote were subject to comparative SC analysis.

**Figure 1.**
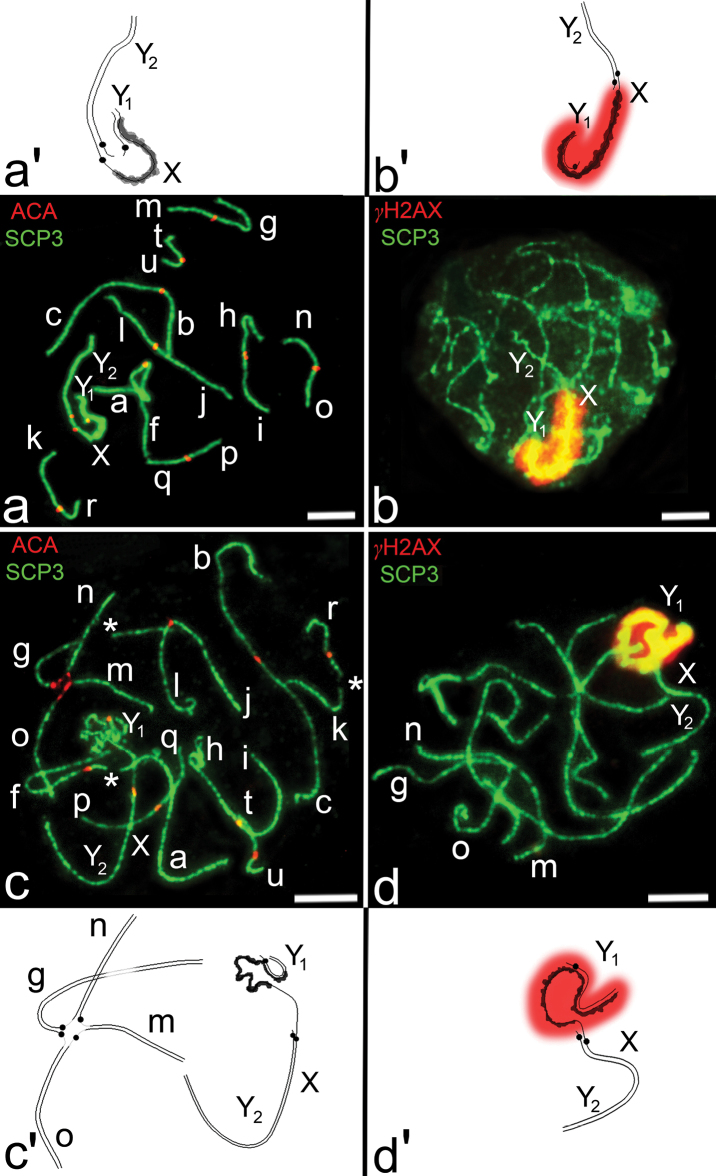
**a–d** Synaptonemal complexes of homozygotes and complex heterozygotes of the common shrew. Immunostaining with antibodies against axial elements of SC - SCP3 (green), polyclonal antibodies to centromeric protein ACA (red) and antibodies to γH2AX (red) marking chromosome asynaptic regions. Bar = 5 μm **a, b** SCs from spermatocyte pachytene nuclei (the Moscow race) **a** Nine SC bivalents (*af*, *bc*, *jl*, *hi*, *gm*, *no*, *kr*, *pq*, *tu*) and sex trivalent XY_1_Y_2_. Sex trivalent contains irregular thickening of the “true” arm of X-chromosome (scheme **a’**). The autosomal arm of the X-chromosome forms a typical SC. Centromeres within *hi* bivalent and XY_1_Y_2_ trivalent are displaced relative to each other **b** Anti-γH2AX antibodies recognize chromatin in the synaptic zone of X and Y_1_ chromosomes and unsynapsed thickened region of the “true” arm of X-chromosome (scheme **b’**) **c, d** SCs from spermatocyte pachytene nuclei obtained from Moscow-Neroosa hybrids **c** Seven SC bivalents (*af*, *bc*, *jl*, *hi*, *kr*, *pq*, *tu*), sex trivalent XY_1_Y_2_ and SC tetravalent (*g/o/n/m*) were revealed in spermatocyte nuclei of complex heterozygotes. Gaps were detected in SC bivalents *af*, *kr* and in *g* arm of SC-tetravalent (indicated with asterisks). Gaps were also detected in pericentromeric regions of all metacentrics of the SC tetravalent (scheme **c’**). *af* SC bivalent is associated with sex trivalent; **d** Anti-γH2AX antibodies identify chromatin in the synaptic region of X and Y_1_ chromosomes and asynaptic thickening of the “true” arm of the X-chromosome (scheme **d’**), as for common shrew spermatocytes from Moscow race (Fig. 2b). One of the SC bivalents is associated with the true part of sex trivalent. The SC tetravalent is usually associated with one or two autosomes (**c, d**).

**SC analysis of a homozygote of the common shrew.** Immunocytochemical analysis of SCs in pachytene spermatocytes of the homozygote revealed nine SC bivalents (*af*, *bc*, *jl*, *hi*, *gm*, *no*, *kr*, *pq*, *tu*) and the sex trivalent (XY_1_Y_2_), as expected from the G-banded karyotype of the Moscow race. Centromeres of *hi* SC bivalent and centromeres in the sex trivalent were not aligned. The sex trivalent exhibited irregular thickenings of the “true” arm of the X chromosome. The autosomal arm of the X chromosome formed a typical SC (Fig. 1a, a’). γH2AX covered only the synaptic region of the X and the Y_1_ chromosomes and the thickened part of the X chromosome. The autosomal arm of the X chromosome is not involved in inactivation (Fig. 1b,b’).

**Figure 2.**
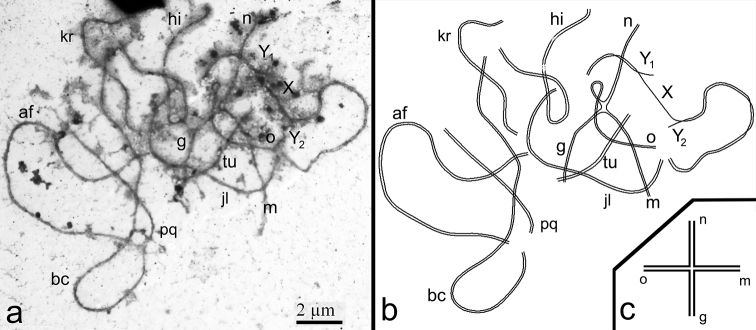
**a–d** A pachytene spermatocyte of the Moscow-Neroosa hybrid. **a** An electron micrograph. Seven SC bivalents (*af*, *bc*, *jl*, *hi*, *kr*, *pq*, *tu*), the sex trivalent XY_1_Y_2_ and the SC tetravalent (*g/o/n/m*) are detected. Closed SC tetravalent is composed of four monobrachial homologous metacentrics *go*, *on*, *nm*, *mg*. SC tetravalent is associated with two autosomes and sex trivalent. Bar = 2 μm **b** A scheme of chromosome synapsis on the basis of Fig. 2a **c** A scheme of SC tetravalent.

**SC analysis of complex heterozygotes of the common shrew.** As expected, seven SC bivalents (*af*, *bc*, *jl*, *hi*, *kr*, *pq*, *tu*), an SC tetravalent (*g*/*o*/*n*/*m*) and the sex trivalent XY_1_Y_2_ were detected in spermatocyte nuclei at pachytene stage (Fig. 1c, c’, 2). According to the previously elaborated classification, the SC tetravalent represents a closed SC multivalent which was formed due to monobrachial homology (Matveevsky and Kolomiets 2011). Arms *af*, *kr* of SC bivalents, the *g* arm and pericentromeric regions of the SC tetravalent contain gaps (Fig. 1c). Sex trivalents were recurrently located at the periphery of the pachytene nuclei of spermatocytes. The “true” X arm of the sex trivalent was irregularly thickened and covered with γH2AX (Fig. 1d, d’).

**Figure 3.**
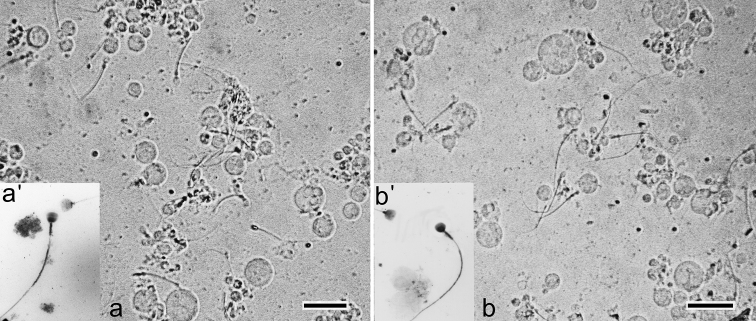
**a–b** Cell suspension of common shrew testis **a** – homozygote (the Moscow race) **b** – complex heterozygote from interracial hybrid zone **a’, b’** Inverted image of spermatozoa (non-specific binding of anti-SCP3 antibodies after immunocytochemistry). Bar = 20 μm.

**Suspension of testis cells.** There are spermatocytes and active spermatozoa in testis cell suspension from common shrews of both Moscow race and hybrids (Fig. 3). Chromosome spreads also contained a significant amount of spermatozoa (Fig. 3a’, b’).

## Discussion

Hybrid zones of *Sorex araneus* represent unique natural laboratories for studying Robertsonian chromosomal polymorphism. Complex cytogenetic studies have been carried out in 17 known chromosomal hybrid zones; however only seven of them have been subjected to analysis of early stages of meiosis including synaptonemal complexes analysis (see Table 1). Such studies provide information about the peculiarities of chromosomal synapsis and separation of multivalents in meiosis, which determine a hybrid’s sterility/fertility. The latter is important for estimation of reproductive isolation level between different chromosomal races.

The model of chromosomal speciation by monobrachial centric fusion has been proposed by Baker and Bickham (1986). Fixation of metacentric chromosomes with monobrachial (single-arm) homology formed as a result of independent fusion of acrocentric chromosomes can entail reproductive isolation of population and further speciation due to the accumulation of genetic differences (Capanna 1982, Baker and Bickham 1986). It is considered that such a mechanism of speciation occurs among some mammal species: lemur genus *Eulemur* Simons et Rumpler, 1989 (Djlelati et al. 1997, Rumpler 2004), bat genus *Rhogeessa* H. Allen, 1866 (Baker et al. 1985), mole vole of the *Ellobius tancrei* (Bakloushinskya et al. 2010), beaver genus *Castor* Linnaeus, 1758 (Ward et al. 1991), mouse of the *Mus musculus domesticus* (Capanna et al. 1976), rat genus *Rattus* Fischer de Waldheim, 1803 (Baverstock et al. 1983). Searle (1988) suggested that monobrachial fusions may contribute to speciation in *Sorex araneus*.

It was assumed that interracial hybrids of the common shrew (complex heterozygotes with multiple Rb rearrangements) had either significantly reduced fertility or were completely sterile (Searle 1988, 1993, Aniskin and Lukianova 1989). Mice and mole voles that differed in several Rb translocations, exhibited reduced fertility too (Capanna 1975, Lyapunova et al. 1990, Hauffe and Searle 1998, Bakloushinskaya et al. 2010). Furthermore, reduced fertility and presence of aneuploid cells were revealed in heterozygotes from different hybrid zones of house mouse chromosomal races that varied in monobrachial homologous metacentrics (Said et al. 1993, Nunes et al. 2011). The fertility of hybrids most likely depends on the amount of monobrachial homologous metacentrics. Indeed, complex heterozygotes of *Sorex araneus* from the contact zone of Oxford and Hermitage races had an increased content of defective testicular tubes and testis with reduced weight, whereas simple heterozygotes were similar to homozygotes (Garagna et al. 1989). However, in the common shrew, even extremely long meiotic chain configurations may not necessarily lead to complete sterility (Mercer et al. 1992). For example, hybrids from a contact zone of Moscow and Seliger races that exhibited the most diverse pattern of monobrachial homologous metacentrics with an additional WART translocation had mature spermatozoa (Pavlova et al. 2008).

Previous studies have also demonstrated that association of autosomes and complex SC configurations with sex chromosomes in meiotic prophase I could cause reduction of fertility or even complete sterility (Forejt et al. 1981, Burgoyne and Baker 1984). It should be noted that, unlike SC trivalents, complex SC configurations are often associated with autosomes and sex trivalents (Narain and Fredga 1997, Pavlova et al. 2008). We also revealed that sometimes the SC tetravalent interacted with the sex trivalent in the complex heterozygotes that we examined. In previous works similar contact sites (or physically interactions) of autosomes, SC multivalents and sex bivalents were interpreted as associations (Mercer et al. 1992, Narain and Fredga 1997).

To reveal the signs of defects in spermatogenesis in our specimens, we studied the dynamics of meiotic prophase I focusing on the sex trivalent. Normally, sex chromosomes of male mammals contain a short SC in pseudoautosomal region and long unpaired axes in meiotic prophase I. Also, sex chromosomes often move to the periphery of pachytene nuclei and undergo MSCI (*meiotic sex chromosome inactivation*), which is required for successful progression of meiosis (Forejt 1984, Burgoyne et al. 2009). We used SCP3 antibodies to identify the SC axial elements and γH2AX antibodies to mark chromosome asynaptic regions. This marker was revealed in chromosome asynaptic regions starting from leptotene and up to late diplotene in cases of incomplete synapsis (Turner et al. 2006).

We found that the behavior of the sex trivalent in homozygotes (Moscow race) was similar to that of sex chromosomes in meiotic prophase I in other mammals. However, in complex heterozygotes, the sex trivalent interacted with autosomes in some prophase nuclei, which was typical of hybrids and heterozygotes with chromosomal rearrangements and reduced fertility (Forejt et al. 1981). Thus, we do not exclude a possibility of partial loss of spermatocytes due to this condition.

Formation of complex SC configurations is known to be associated with a high degree of asynapsis. In such cases, chromosome asynaptic regions undergo transcriptional inactivation MSUC (*meiotic silencing of unsynapsed chromatin*), which in its turn results in meiotic arrest and reduction of fertility (Homolka et al. 2007, Mahadevaiah et al. 2008). Nonetheless, no MSUC signs were detected in the hybrids of *Sorex araneus*. In the gap regions (SC tetravalent, *af*, *kr* SC bivalents), γH2AX was not detected. Most probably, gaps in pericentromeric regions of the SC tetravalent do not reflect asynaptic regions, but may result from the extension of chromosome axial elements due to the alteration of nucleus organization and retention of telomere links with nuclear envelope. Similar trends in tetravalent dynamics were revealed in the progeny of radiation-exposed male mice (Kolomiets et al. 1992). Closed SC multivalents associate with the sex bivalent to a lesser extent and therefore do not cause meiotic failures.

Probably, the four metacentrics that form SC tetravalent in interracial Moscow-Neroosa hybrids undergo successful separation, spermatocytes are not arrested (or are arrested partially) and balanced gametes are formed in the end. This is also supported by the presence of numerous spermatozoa in hybrid testis cell suspensions. Further studies are needed to measure the level of aneuploidy.

Our data conform to the results of other authors. For example, no defects of sex body formation were detected in most spermatocyte nuclei of mice that were heterozygous for eight Rb translocations, which indicated moderate activity of pachytene arrest (Manterola et al. 2009). Association of the SC trivalent carrying asynaptic regions with the XY bivalent in early-middle pachytene, which also did not result in the reduction of fertility, was revealed with immunocytochemical methods in laboratory mice with a single translocation (Saferali et al. 2010). Apparently, in case of Rb translocations the reduced efficiency of checkpoint in pachytene determines the possibility of Rb metacentric circulation in natural populations and their role in karyotype evolution (Matveevsky and Kolomiets 2011).

In our study, centromeres of homologues in the SC bivalent formed between two Rb metacentrics (*hi*) were not aligned. We assume that this pattern might result from different mechanisms of Rb metacentric formation in the past. One of the ancestors might have retained centromere of *h* chromosome after formation of Rb metacentric, while another ancestor might have retained centromere of *i* chromosome. Previous works reported a presence of two centromeric foci in other Rb metacentrics (Borodin et al. 2008). We suggest that in case of extensive chromosomal variability of *Sorex araneus*, centromere polymorphism of Rb metacentrics might be often observed in different populations.

Our synaptonemal complex results suggest regularity in formation of the ring-of-four configuration produced by hybrids between the Moscow and the Neroosa chromosome races of *Sorex araneus*. This relates well to the previous findings that chromosome races differing by monobrachial homology in *Sorex araneus* does not lead to complete sterility in hybrids. In particular, our immunocytochemical demonstration of an absence of asynapsis in the ring-of-four configuration relates well to the production of sperm in such hybrids.

**Table 1.** SC analysis in chromosomal hybrid zones of the common shrew.

**Hybrid zone**	**Examined karyotypic categories**	**Detected SC-configuration**	**Reference**
Oxford Hermitage	SH (chain-of-three) *(k/q), (n/o), (p/r)*	SC trivalents	Wallace and Searle 1990
Oxford Wirral	SH (chain-of-three) *(k/q), (n/o), (k/o)*, *(j/l)*	SC trivalents	Borodin et al. 2008
Abisko Sidensjö	CH (chain-of-four) *i*/*ih*/*hn*/*n*	SC tetravalent	Narain and Fredga 1998
Aberdeen/ Oxford	CH (chain-of-seven) *r*/*rp*/*pn*/*no*/*ok*/*kq*/*q*	SC chain with 7 elements	Mercer et al. 1992
Novosibirsk/Tomsk	CH (chain-of-eight) + (chain-of-three) *o*/*og*/*gk*/*ki*/*ih*/*hn*/*nm*/*m, q/r*	SC chain with 8 elements and SC trivalent	Karamysheva et al. 2007
Moscow Seliger	CH (chain-of-eleven) *g*/*gm*/*mh*/*hi*/*ik*/*kr*/*rp*/*pq*/*qn*/*no*/*o*	SC chain with 11 elements	Pavlova et al. 2008
Uppsala Hällefors	CH (ring-of-four) *qp*/*pk*/*ko*/*oq*	SC tetravalent	Narain and Fredga 1997
Moscow Neroosa	CH (ring-of-four) *og/gm/mn/no*	SC tetravalent	this study

SH – simple heterozygotes, CH – complex heterozygotes
